# Food Safety Knowledge, Attitude, and Practice of College Students, Ethiopia, 2019: A Cross-Sectional Study

**DOI:** 10.1155/2021/6686392

**Published:** 2021-01-12

**Authors:** Jember Azanaw, Henok Dagne, Zewudu Andualem, Tsegaye Adane

**Affiliations:** Department of Environmental and Occupational Health and Safety Institute of Public Health, College of Medicine and Health Sciences, University of Gondar, Gondar, Ethiopia

## Abstract

**Background:**

Since the dawn of human history, foodborne diseases have been a problem for all societies, and it is an increasing public health issue worldwide. The objective of this study is to examine knowledge, practice, and attitude in food safety among college students in the city Gondar, northwest Ethiopia.

**Methods:**

A cross-sectional study was conducted among 430 randomly selected college students in Gondar City, northwest Ethiopia, from October 8 to November 30, 2019. The data was gathered through a self-administered questionnaire. The collected data were checked for completeness, coded manually, and entered into Epi Info version 7, then exported to SPSS version 26 for more data checking, cleaning, and analysis. One-way ANOVA was done for examining sociodemographic variable association with KAP, and Pearson correlation analysis was conducted to measure the association among food safety knowledge, attitude, and practice scores. The *p* value < 0.05 was considered statistically significant for both tests.

**Results:**

The number of females was higher among the study participants (65.5%). The mean age of participates was 21.1 ± 9.14 (SD) years. The higher percentage (45.7%) of the participants were students in the second year. The overall scores of the food safety knowledge, attitude, and practices of the respondents were 35.7% (good), 38.0% (good), and 29.1% (positive), respectively. Participants with differences in gender, year of study, and food safety training had a substantial variation in overall food safety knowledge (*p* value < 0.05). The findings have clearly shown that knowledge of food safety does not directly affect the attitude towards food safety (*p* value > 0.05). On the other hand, overall food safety practice differed in terms of sex, year of schooling, and knowledge of food safety (*p* value < 0.05) among participants. Training and knowledge score had significant correlation among respondents (*p* value < 0.05).

**Conclusion:**

This study revealed that the overall knowledge, practices, and attitude towards food safety among college students were very low. There was a significant association between knowledge and practice while food safety practices were independent with an attitude among the students. Such results indicated that there could be problems with foodborne diseases. Therefore, the findings of this study proposed that strength should be done to improve the existing food safety knowledge, practice, and attitude in college students in addition to their normal education.

## 1. Background

Since the dawn of human history, foodborne diseases (FBD) have been a problem for all societies and are a growing public health issue worldwide [1–3]. Foodborne illnesses are serious and persistent issues that lead to extreme morbidity and often death [4, 5]. Millions of people are expected to become ill each year, and thousands of them die after eating contaminated food due to poor food handling [6]. About half of the global foodborne disease burden is accounted for by diarrhoeal diseases, causing 550 million people to get sick and 230,000 deaths every year [6]. In 2010, the burden of foodborne diseases was 33 million safe years lost (DALY), 600 million foodborne diseases, and 420,000 deaths, of which foodborne diseases were diarrhoeal [5].

In less developed countries, however, the occurrence of foodborne disease is more prevalent due to decreased sanitation, lack of clean drinking water, polluted inadequate food storage facilities, and lack of food safety education [7]. Despite several studies and action plans in the field of food protection, in nondeveloped countries, more than 91 million people are affected by foodborne diseases [8]. Real epidemiological data on foodborne diseases, however, continue to be scarce, especially in these countries, including Ethiopia [9]. Since the problem of foodborne diseases is universal, it therefore requires the cooperation, funding, understanding, and promises of all stakeholders from different governments and policy makers. The responsiveness and teaching of food safety in the populations should be emphasized and promoted [10]. Food handlers and consumers are essential components of foodborne disease outbreaks due to food mishandling by planning, processing, and storage [11]. Nonetheless, there are various factors affecting the rate of foodborne diseases in unindustrialized nations that can lead to a reduced occurrence of these diseases adequately addressed [12]. The knowledge, attitude, and practice of food handlers towards food safety are factors in sustenance poisoning [13].

Outbreaks of foodborne diseases occur at home and at group social gatherings [10]. The lack of knowledge of food safety, decreased personal hygiene, improper handling of food, and household preparation in developing countries serve as main contributors to outbreaks of foodborne diseases due to contamination of raw food with processed food [14]. Household preparations, in due course, play an important role in foodborne illnesses [15].

Therefore, recognizing the food safety knowledge, attitudes, and practices of food handlers is critical in finding ways to minimize the risk of foodborne diseases [16, 17]. Food safety education is supportive for young people because it would be appropriate to establish positive attitudes and good knowledge and practices to increase current food safety issues [18]. And there is a critical need to examine the knowledge, attitude, and practice of undergraduate students on food safety in their potential roles for a healthier generation [15]. Several studies have also recorded low food safety awareness and practice among students in higher education institutions [15, 19–21]. Scholars suggested that more KAP studies on food poisoning are needed for various target residents, particularly in less developed countries [22]. Nevertheless, most food safety studies in Ethiopia have been carried out in public food establishments [23–28] but have not carried out knowledge, attitudes, and practices among students of the higher education class. The importance of this research is enhanced by all the above and other issues. Therefore, the main objective of this study was to study the level of knowledge, practice, and attitude among college students in Gondar city, northwest Ethiopia.

## 2. Method

### 2.1. Study Design, Period, and Setting

A cross-sectional study was carried out from 8 October until 30 November 2019 in two randomly selected college students, in Gondar City. Gondar City is located in Central Gondar Zone, northwest Amhara region, Ethiopia. It is located at 748 km from Addis Ababa (the capital city of Ethiopia) and 180 km from the capital of the Amhara region, Bahir Dar district. There are four public colleges in Gondar City with different disciplines.

### 2.2. Sample Size Determination and Sampling Strategy

The sample size was calculated based on the assumption of a 50% proportion (*p*) (because there is no similar study in the study area), 5% estimated margins of error (*d*), and 95% confidence interval (*Zα*/2) and 12% dropout: *n* = ( *Z*_*a*/2_)^2^*P*(1 − *P*)/*d*^2^, *n* = ( 1.96)^2^0.50(1 − 0.50) /0.05^2^, *n* = 384 + 46 = 430.

Then, for the analysis, the final sample size required was 430. Two schools were selected using lottery methods from four public colleges. The number of students needed in each year was equivalently calculated by sample size calculation on the basis of the number of students before the representative sample was touched. Then, research participants were chosen by means of stratified random sampling by selecting the four student groups based on the year of study. Students involved in this research were chosen from those who were studying in various departments from 1^st^ to 4^th^ years.

### 2.3. Data Collection Tools and Procedures

In order to evaluate the food safety knowledge, attitude, and food practices of college students in Gondar city, a questionnaire was developed. A self-administered organized survey containing four investigation subsections was designed by investigators. Sociodemographic characteristics such as gender, age, marital status, department, year of study, resident, family size, monthly income, and number of educated families were the first section. The second subsection of the questionnaire includes 10 questions about the knowledge of students in the field of food safety. This was replied yes/no and do not know, by multiple choices. The answers were registered as one for correct responses and zero for incorrect responses/do not know responses [29]. Based on Bloom's cut-off points, the KAP among study participants was grouped into three levels, 80.0%-100.0%, 60.0–79.9%, and <60.0% [23, 30]. The knowledge records were then divided into three statuses: 8-10 scores good, 6-7 scores fair, and 0-5 scores poor. The third portion of the questionnaire consisted of 20 questions about the participants' food safety practices. Questions were used to calculate the 5-point Likert scale in practice with choices from “never” to “always,” but the scale was reversed for the negative statements during recod. Total practice data was graded as good for 80 to 100 points, moderate for 60 to 79 scores, and poor for less than 60 scores. The fourth subsection was questioned for an attitude regarding food safety. Thirteen questions with five-response choices from “strongly disagree” to “strongly agree” were used to ask these concerns. Questions in derogatory sentences reversed the order of the ratings. The responses were categorized into three levels, such as 52-65 positive attitude, 39-51 neutral attitude, and <39 negative in food safety attitude. The questionnaire also includes an introductory part that explains the study's purpose, the voluntarism of participants, and the time needed to complete the study.

### 2.4. Data Quality Management

The questionnaires were constructed by reviewing previous comparable research [19, 31–34]. To gain content validity, the questionnaire was first prepared in English and then translated into Amharic (local language) and back to English. In order to determine its simplicity, the appropriateness of the language, and the average time needed for completion, the 22 questionnaires were pretested at the earliest. The reliability test was tested for all knowledge, attitude and practice, questions, and values using Cronbach's alpha 0.73, 0.85, and 0.71, respectively. These results showed that all the items were internally consistent and reliable [25]. Data gained from the pretest were not incorporated in the last analysis. Depending on the pretest, essential adjustments of the questionnaire were recognized and addressed through expert consultation. The questionnaire took nearly 20 minutes to finish. The supervisors assured data quality through close monitoring during collection, checking each of the completed questionnaires regularly, and refilling out those questionnaires that were not completed. Researchers engaged in supervising, data cleaning, entry, analysis, and writing up.

### 2.5. Data Collection

The survey protocol, questionnaire, and informed agreement were revised and accepted by the Review Committee of the Department of Environmental and Occupational Health and Safety (Ref. No. EOHS/174/2019). Before collecting the data, a letter was issued for school deans clarifying the objectives and for asking their cooperation in doing so. Enrolment for the survey was through the help of assigned students and teachers. These individuals distributed questionnaire to individuals who were involved in joining the study. Before starting the data collection, study participants were asked for verbal consent and expressed that they can be left to participate in this study. This means that all study participants were included with their willingness. Then surveys were done and collected before class, when there is no class, during break time, and after class immediately as the teachers ended their period. Students who did not volunteer to participate were excluded from this study.

### 2.6. Data Processing and Analysis

Data obtained during the survey were coded, entered into Epi Info version 7, and analyzed in SPSS (Statistical Package for the Social Sciences) software version 26.0. Descriptive (frequency, standard deviation, median, and mean) statistics were used to report results from the study. One-way ANOVA was done for examining sociodemographic variables with KAP, and Pearson correlation analysis was conducted to measure the association among food safety knowledge, attitude, and practice scores. For all analyses, *p* value < 0.05 was considered statistically significant.

## 3. Results

### 3.1. Sociodemographic Characteristics of the Participants

A total of 430 students included in this study have a response rate of 99.77% (429/430). Females were the largest number (65.5%) among the total participants, while 34.5% (148/429) were males. The mean age was 21.1 ± 9.14 (SD) years, and 42.0% of the respondents were less than 19 years. The majority (90.4%) of respondents were unmarried, and two hundred seventy-two (63.4%) came from urban residents. The higher proportion of participants (45.7%) were second-year students (Table 1).

### 3.2. Food Safety Knowledge of the Participants

From 10 knowledge-related questions, 153 (35.7%) of the study participants answered more than 80%, 28.2% from 60%-79.9%, and 36.1% less than 60%, which showed that the majority of the study participants had a low level of knowledge in food safety. In the current report, 71.3% of participants recognized cross-contamination by the interaction between cooked and noncooked foods in food safety activities, but only 42.4% of respondents knew that using the same knife to cut vegetables and meat exposed them to foodborne diseases. Most of the students in this study battled over the forms in which food safety was maintained, such as wearing a glove to avoid food contamination (63.2%) and regular washing of food contact surfaces to prevent food contamination (84.6%). The correct way to wash hands is practiced by just a few study participants (29.6%). The majority (94.9%) of respondents had knowledge about the temperature danger zone for food contamination. Respondents had good knowledge of contaminated food detection, with 83.4% reporting that sensory organs could not be used to detect food contamination (Table 2).

### 3.3. Practices towards Food Safety

Out of 100, the overall score was for food safety practice. Thirty point eight percent (132/429) had good, 39.6% moderate, and 29.6% poor practice in food safety. In these graduate students' evaluation of food safety procedures, only 32.6% recorded that they always wash their hands with soap and water before handling and preparing the food. More than half (52.9%) of respondents never used a thermometer to assess whether or not the food was cooked thoroughly. Just a few (14.2%) use thermometers to calculate the refrigerator temperature. One hundred thirty-three (31.0%) of the participants in the study always verify the selling and use of food materials by dates (Table 3).

### 3.4. Attitude in Food Safety

Among the study participants, 29.1% have positive, 37.3% neutral, and 33.6% negative attitude in food safety. One-fourth (25.0%) of respondents accepted that covering the mouth and nose while coughing and sneezing would avoid food contamination. Participants also decided (35.4%) that individuals with abrasions or cuts on their hands should not touch ready food. One hundred thirty-one (30.7%) of the respondents disagreed that touching food barehanded contributes to the contamination of food. In addition, more than half of the study subjects (54.1%) disagreed that it is necessary to maintain personal hygiene during food preparation to control foodborne diseases. About 50.8% of respondents had a good outlook about pathogens contaminating food with long and painted fingernails (Table 4).

### 3.5. Association between Sociodemographic Variables and KAP of Study Participants

Analysis using one-way ANOVA revealed that there was statistically significant variation when comparing the practice of respondents with their sex. The ANOVA test showed that concerning practice towards food safety, there is a statistically significant difference among their year of education. And also there was a significant difference among participants in their overall food safety knowledge and practice with the year of study (*p* value = 0.010) and (*p* value = 0.002), respectively. The other way of comparison was participants' KAP and their department. However, here, only knowledge showed the variation (*p* value = 0.009) regarding the participant department. One-way ANOVA showed that there was a statistically substantial difference in knowledge by groups in food safety-related training, but there is no variation in practice and attitude (Table 5).

### 3.6. Pearson Correlation Analysis Average at Food Safety Knowledge, Practice, and Attitude

The results of correlation analysis indicated that a significant difference was found in the food safety practice of the participants who had poor and good food safety knowledge (*p* value < 0.05). However, there is no significant difference between practices among respondents due to variations in their attitude (Table 6).

## 4. Discussion

In industrialized and developing nations, foodborne illnesses are characterized as prevalent and rising public health issue [22]. This study focused on evaluating food safety knowledge, practices, and attitude among college students at Gondar city which play a major role in foodborne illness reduction.

### 4.1. Knowledge towards Food Safety

In the current study, only 35.7% of the participants were good in overall food safety knowledge. This was lower than other related studies conducted in Malaysia [35], Bulgaria [14], and Tehran [36], although it is greater than the study conducted in Teheran [37]. This finding is in line with other findings done in Beijing [21] and Ankara [38]. This variation might be due to their difference in their sociodemographic characteristics, study time, study setting, and study design. Studies indicated that there is an increase in knowledge about food safety as educational status escalates [22, 39]. Also, this finding showed that a statistically significant difference in students' food safety knowledge according to their difference in year of education, department, and food safety-related training (Table 5). With regard to individual questions, the majority (85.8%) of the respondents understand that contaminated foodstuffs always change their characteristics, and less than three-quarters (42.4%) of them know that using the same knife in cutting vegetables and meat exposed them to foodborne diseases. This is the major cause of cross-contamination in food handling. Only 17.0% of the students had knowledge that uncooked meat should be stored in the lower part of the refrigerator. Around ninety-five percent (94.9%) of students know the danger zone for food safety (40-140°F) (Table 2).

### 4.2. Practice regarding Food Safety

30.8% of respondents had good practice towards food safety. This finding is lower than that of other similar studies [14, 22, 38]. In this study, there was no statistically significant correlation between sex differences, but other studies showed that sex among participants was significantly associated with food safety practices [14, 15, 20, 40, 41]. But this finding is consistent with other studies done at the University of Bulgaria [14] and Palestine [42]. There was a practice difference in food safety among respondents regarding the level of knowledge. This was supported by other studies that showed that poor food safety practices of food handlers are explained by the lack of knowledge and good food safety practices for those participants related to good knowledge [11, 43–47].

This section focused mainly on food safety practices, including cross-contamination prevention, temperature determination, and personal hygiene, especially hand hygiene practices, since improper handling of food is a major cause of foodborne illness and poor hand hygiene is a significant risk factor for contamination of food [48]. Food handlers should therefore always wash their hands at any point in food production, in particular before handling food, after feeding, and after touching contaminated materials [49]. In this finding, however, only a few respondents (32.6%, 43.4%, 36.1%, and 43.1%) still wash their hands with soap and water before handling food, after touching dirty products, after using toilets, and handling waste, respectively. It is mandatory to use food temperature measurement instruments such as thermometers to assess if the food is in the danger zone or not [50]. Studies pointed out that the main aim of cooking is to increase food palatability, to destroy bacteria, and to increase the protection and storage life of the foodstuffs [51, 52]. However, 44.3% and 52.9% of participants never used thermometers in this study to determine the temperature of the refrigerator and thoroughly cooked foods, respectively (Table 3). Approximate temperature exposes to 50% of foodborne disease in food storage/reheating and 45% associated with inappropriate food storage temperature in food storage [42]. These all might be public health problems related to foodborne diseases.

### 4.3. Attitude towards Food Safety

Food safety attitude is a crucial aspect that may influence food safety performance and practice, accordingly lessening the happening of foodborne illnesses [11]. However, in this study, from the overall attitude questions, only 29.1% of the study participants had a positive attitude toward food safety. It was lower than findings conducted in Taif University students [22], students in Shahroud University [37], students at Trakia University, Bulgaria [14], and private university students, Malaysia [53]. This difference might be due to their dissimilarity in their sociodemographic profile, study period, study area, and study design. In the current study, there were significant differences in attitudes towards food safety due to their variation in the level of knowledge (Table 6). This finding was supported by other studies, which revealed that there was a significant association between knowledge level and attitude score. However, this result was contradicted by another study [54]. On the other hand, there was no substantial correlation between food safety attitude and food safety practice. This finding was in line with another study conducted in Malaysia [54]. Other studies have shown that positive attitudes motivate food handlers to have a greater effect on their food safety practices [55], and it is more significant than knowledge and practice [56]. Only three-quarters of the students agree that covering the mouth during coughing and sneezing avoids contamination of food. More than half (56.4%) of respondents did not believe that proper hand hygiene can prevent foodborne diseases. A consequence of low hand washing is the existence of dangerous microbes on hands by touching uncooked food elements and other materials [57]. Around one-fourth (27.7%) and 47.3% of the participants were neutral and disagree, respectively, that “covering mouth during coughing and sneezing prevents food infection” (Table 4). However, sneezing and coughing can release dewdrops of fluid and possibly infectious microbes which moves up to 7-8 meters [58, 59]. Finally, such low attitudes of the respondents lead to foodborne diseases, because attitude is inherent that enforces good food safety handling.

### 4.4. Limitations of the Study

This research typically takes into account certain limitations when evaluating the findings. The first was the inherent limitation of the nature of the cross-sectional sample that does not allow cause-effect relationships to be formed, and second, social desirability bias may occur. Furthermore, because it was self-reported, this study was not supported by observation, particularly for practices.

## 5. Conclusion

This study revealed that the overall knowledge, practices, and attitude towards food safety among college students were very low. There was a significant association between knowledge and practice while food safety practices were independent with an attitude among the students. Such results indicated that there could be problems with foodborne diseases. Therefore, the findings of this study proposed that strength should be done to more improve the existing food safety knowledge, practice, and attitude in college students in addition to their normal education.

## Figures and Tables

**Table 1:** Sociodemographic profile of study participants (*n* = 429).

Variables		Frequency (*n*)	(%)
Sex	Male	148	34.5
	Female	281	65.5
Age in years	<19	180	42.0
	19-20	82	19.1
	20.5-22	67	15.6
	>22	100	23.3
Mean age = 21.1 ± 9.14 (Std. D)			
Income level (*n* = 222)	<2000	85	19.8
	2000-3000	37	8.6
	3001-5000	52	12.1
	>5000	48	11.2
Marital status	Married	41	9.6
	Unmarried	388	90.4
Residence (*n* = 420)	Rural	148	34.5
	Urban	272	63.4
Educational level	First year	144	33.6
	Second year	196	45.7
	Third year	57	13.3
	Fourth year	30	7.0
Department	Food preparation	101	23.5
	Hotel management	125	29.1
	Tourism	122	28.4
	Other	81	18.9
Training-related food safety (*n* = 426)	Yes	165	38.5
	No	261	60.8
Foodborne diseases within the past two weeks	Yes	41	9.6
	No	388	90.4

**Table 2:** Knowledge in food safety among participants.

No.	Information concerned with food safety	Incorrect	Correct
		*N* (%)	*N* (%)
(1)	Uncooked meat should be stored in the lower part of the refrigerator	356 (83.0)	73 (17.0)
(2)	Contact between cooked and uncooked foods causes cross-contamination	123 (28.7)	306 (71.3)
(3)	Wearing gloves will reduce the contamination of food	158 (36.8)	271 (63.2)
(4)	Contamination of foodstuffs cannot be detected using sense organs	71 (16.6)	358 (83.4)
(5)	Followed the proper form of procedure for washing hands	302 (70.4)	127 (29.6)
(6)	Followed the correct method for washing food equipment	347 (80.9)	82 (19.1)
(7)	Use of the same knife to cut vegetables and meat exposed to foodborne diseases	247 (57.6)	182 (42.4)
(8)	Contaminated foodstuffs always change their characteristics	61 (14.2)	368 (85.8)
(9)	Food contamination risk zone (40-140°F)	22 (5.1)	407 (94.9)
(10)	Frequent food contact surface cleaning can prevent contamination of the food	66 (15.4)	363 (84.6)
The overall knowledge	Good	153	35.7
	Fair	121	28.2
	Poor	155	36.1

**Table 3:** Participants' food safety practice.

Questions on food safety-related activities	Never	Rarely	Sometimes	Most of the time	Always
	*N* (%)	*N* (%)	*N* (%)	*N* (%)	*N* (%)
(1) Do you wash your hands before handling and cooking food with soap and water	39 (9.1)	80 (18.6)	88 (20.5)	82 (19.1)	140 (32.6)
(2) Do you cover your hands with cut/sore before preparing food	52 (12.1)	69 (16.1)	76 (17.7)	63 (14.7)	169 (39.4)
(3) Do you wash your hands with soap and water after you touch the raw meat, poultry, and seafood	13 (3.0)	55 (12.8)	79 (18.4)	93 (21.7)	189 (44.1)
(4) Before using cooked food, you wash plates used for raw meat, poultry, and seafood	32 (7.5)	57 (13.3)	85 (19.8)	85 (19.8)	170 (39.6)
(5) Using the same cutting board for raw meat, poultry, seafood, and vegetables	95 (22.1)	80 (18.6)	59 (13.8)	80 (18.6)	115 (26.8)
(6) Using frozen meat thawed in the morning to cook in the evening	77 (17.9)	69 (16.1)	84 (19.6)	70 (16.3)	129 (30.1)
(7) Left cooked food near the counter, to be used the next day	77 (17.9)	62 (14.5)	103 (24.0)	81 (18.9)	106 (24.7)
(8) Store eggs at room temperature	106 (24.7)	61 (14.2)	88 (20.5)	77 (17.9)	97 (22.6)
(9) Using hot and soapy water after food preparation to disinfect countertops	59 (13.8)	68 (15.9)	69 (16.1)	88 (20.5)	145 (33.8)
(10) To determine the temperature of the refrigerator, use the thermometer	190 (44.3)	59 (13.8)	71 (16.6)	48 (11.2)	61 (14.2)
(11) Using a thermometer to assess if the food is fully cooked	227 (52.9)	51 (1.9)	52 (12.1)	51 (11.9)	48 (11.2)
(12) To decide whether leftovers have been reheated, use a thermometer	48 (11.2)	51 (11.9)	52 (12.1)	51 (11.9)	227 (52.9)
(13) Eating runny yolk eggs or items containing crude eggs	73 (17.0)	57 (13.3)	57 (13.3)	67 (15.6)	175 (40.8)
(14) After 3-4 days, should you discard refrigerated leftovers	49 (11.4)	65 (15.2)	77 (17.9)	83 (19.3)	155 (36.1)
(15) Check sale by and used by dates when you buy foodstuffs	101 (23.5)	54 (12.6)	74 (17.2)	67 (15.6)	133 (31.0)
(16) Clean your hands before cooking foodstuffs	49 (11.4)	65 (15.2)	77 (17.9)	83 (19.3)	155 (36.1)
(17) Do you wash your hands before washing the utensils of food	19 (4.4)	47 (11.0)	66 (15.4)	94 (21.9)	203 (47.3)
(18) Do you wash your hands after the toilet	35 (8.2)	47 (11.0)	79 (18.4)	83 (19.3)	185 (43.1)
(19) After counting money, do you wash your hands?	65 (15.2)	68 (15.9)	83 (19.3)	90 (21.0)	123 (28.7)
(20) After handling dirty things, do you wash your hands?	36 (8.4)	58 (13.5)	71 (16.6)	78 (18.2)	186 (43.4)
Overall practice	Good			132	30.8
	Moderate			170	39.6
	Poor			127	29.6

**Table 4:** The participants' attitude towards food safety.

No.	Food safety-related attitude	Agree	Neutral	Disagree
		*N* (%)	*N* (%)	*N* (%)
(1)	Covering the mouth during coughing and sneezing avoids contamination of food	107 (25.0)	119 (27.7)	203 (47.3)
(2)	Do you prefer wearing a ring or watch inside the cafeteria	156 (36.4)	159 (37.0)	114 (26.6)
(3)	Touching food manually without gloves induces food contamination	171 (39.9)	126 (29.4)	131 (30.7)
(4)	After touching some parts of your body, the food handler should wash his hands	121 (28.2)	74 (17.2)	234 (54.6)
(5)	Raw food should be processed separately from cooked food	119 (27.7)	77 (18.0)	233 (54.3)
(6)	Long and painted fingernails contaminate foodstuffs with pathogens	123 (28.7)	88 (20.5)	218 (50.8)
(7)	Proper hygiene of the hand can prevent foodborne illnesses	135 (31.5)	52 (12.1)	242 (56.4)
(8)	Pathogens can be sourced from food utensils	133 (31.0)	76 (17.7)	220 (51.3)
(9)	Food handlers with abrasions on hand do not treat food ready	152 (35.4)	81 (18.9)	196 (45.7)
(10)	Before jobs, the health condition of food handlers should be assessed	128 (59.2)	74 (17.2)	277 (52.9)
(11)	To control foodborne diseases, it is necessary to maintain personal hygiene	143 (33.3)	54 (12.6)	232 (54.1)
(12)	Hands are where most bacteria and microorganism originate	128 (29.9)	74 (17.2)	227 (52.9)
(13)	Left cooked food for more than 2 hours from the refrigerator is unsafe	166 (38.7)	132 (30.8)	131 (30.5)
Overall attitude		Positive	125	29.1
		Neutral	160	37.3
		Negative	144	33.6

**Table 5:** Association of sociodemographic variables and KAP of study participants.

Variables	Mean square	*F*	*p* value
Sex vs. practice	2.885	4.812	0.029
Sex vs. knowledge	0.712	0.990	0.320
Sex vs. attitude	1.125	1.800	0.180
Year of education vs. practice	8.891	5.090	0.002
Year of education vs. knowledge	8.158	3.855	0.010
Year of education vs. attitude	3.745	2.006	0.112
Dept. vs. practice	1.501	0.826	0.480
Dept. vs. knowledge	8.198	3.874	0.009
Dept. vs. attitude	1.921	1.022	0.383
Food safety-related training vs. practice	0.294	0.487	0.485
Food safety-related training vs. knowledge	12.546	18.189	0.0001
Food safety-related training vs. attitude	1.276	2.043	0.154

**Table 6:** Correlation of food safety knowledge, attitude, and practice level.

Variables		Food safety practice	Food safety knowledge	Food safety attitude
Food safety practice	Pearson correlation	1	0.120^∗^	0.054
	Sig. (2-tailed)		0.013	0.265
	Sum of squares and cross-products	258.942	34.023	14.221
	*N*	429	429	429
Food safety knowledge	Pearson correlation	0.120^∗^	1.017	
	Sig. (2-tailed)	0.013		0.724
	Sum of squares and cross-products	34.023	307.991	4.911
	*N*	429	429	429
Food safety attitude	Pearson correlation	0.054	0.017	1
	Sig. (2-tailed)	0.265	0.724	
	Sum of squares and cross-products	14.221	4.911	268.159
	*N*	429	429	429

## Data Availability

The data and materials that are used in this study are available from the corresponding author.

## Abbreviations

DALY: Disability-adjusted life year
FBD: Foodborne diseases
KAP: Knowledge, attitude, and practice
SD: Standard deviation.

## References

[1] Mallach E., Ferrao T., MacLean R., Kirk S. (2016). *Public Health 2016: Time for a Cultural Shift in the Field of Public Health-HPCDP: Volume 36-11*.

[2] Cheraghi Z., Okhovat B., Doosti Irani A. (2014). Knowledge, attitude, and practice regarding food, and waterborne outbreak after massive diarrhea outbreak in Yazd Province, Iran, summer 2013. *International Scholarly Research Notices*.

[3] Lam H.-M., Remais J., Fung M.-C., Xu L., Sun S. S.-M. (2013). Food supply and food safety issues in China. *The Lancet*.

[4] Latif N. A., Elkarmalawy E. M., Esmail G. M. (2013). Impact of food safety educational program on food handlers' knowledge and practice in Cairo governorate. *Journal of American Science*.

[5] Havelaar A. H., Kirk M. D., Torgerson P. R. (2015). World Health Organization global estimates and regional comparisons of the burden of foodborne disease in 2010. *PLoS Medicine*.

[6] Organization WH (2015). *WHO’s First Ever Global Estimates of Foodborne Diseases Find Children under 5 Account for Almost One Third of Deaths*.

[7] Sanlier N. (2009). The knowledge and practice of food safety by young and adult consumers. *Food Control*.

[8] Fukuda K. (2015). *Food Safety in a Globalized World*.

[9] Organization WH (2015). *WHO estimates of the global burden of foodborne diseases: foodborne disease burden epidemiology reference group 2007-2015*.

[10] Takeda S., Akamatsu R., Horiguchi I., Marui E. (2011). Relationship among food-safety knowledge, beliefs, and risk-reduction behavior in university students in Japan. *Journal of Nutrition Education and Behavior*.

[11] Sani N. A., Siow O. N. (2014). Knowledge, attitudes and practices of food handlers on food safety in food service operations at the Universiti Kebangsaan Malaysia. *Food Control*.

[12] Odeyemi O. A. (2016). Public health implications of microbial food safety and foodborne diseases in developing countries. *Food & Nutrition Research*.

[13] Patil S. R., Cates S., Morales R. (2005). Consumer food safety knowledge, practices, and demographic differences: findings from a meta-analysis. *Journal of Food Protection*.

[14] Stratev D., Odeyemi O. A., Pavlov A., Kyuchukova R., Fatehi F., Bamidele F. A. (2017). Food safety knowledge and hygiene practices among veterinary medicine students at Trakia University, Bulgaria. *Journal of Infection and Public Health*.

[15] Sanlier N., Konaklioglu E. (2012). Food safety knowledge, attitude and food handling practices of students. *British Food Journal*.

[16] Majowicz S. E., Diplock K. J., Leatherdale S. T. (2015). Food safety knowledge, attitudes and self-reported practices among Ontario high school students. *Canadian Journal of Public Health*.

[17] Harun H., Abd Samad N., Mohd Hassan N., Abdul Rahman A. W. (2014). *Relationship between Knowledge and Practice among Food Handlers in Vocational College Johor State: Does it Compliance with Food Safety Standards Guide*.

[18] Gavaravarapu S. R. M., Vemula S. R., Rao P., Mendu V. V. R., Polasa K. (2009). Focus group studies on food safety knowledge, perceptions, and practices of school-going adolescent girls in South India. *Journal of Nutrition Education and Behavior*.

[19] Osaili T. M., Obeidat B. A., Jamous D. O. A., Bawadi H. A. (2011). Food safety knowledge and practices among college female students in north of Jordan. *Food Control*.

[20] Hassan H. F., Dimassi H. (2014). Food safety and handling knowledge and practices of Lebanese university students. *Food Control*.

[21] Cheng Y., Zhang Y., Ma J., Zhan S. (2017). Food safety knowledge, attitude and self-reported practice of secondary school students in Beijing, China: a cross-sectional study. *PloS One*.

[22] Sharif L., Al-Malki T. (2010). Knowledge, attitude and practice of Taif University students on food poisoning. *Food Control*.

[23] Gizaw Z. (2014). Food Safety Practice and Associated Factors of Food Handlers Working in Substandard Food Establishments in Gondar Town, Northwest Ethiopia, 2013/14. *International Journal of Food Sciences and Nutrition Diet*.

[24] Azanaw J., Gebrehiwot M., Dagne H. (2019). Factors associated with food safety practices among food handlers: facility-based cross-sectional study. *BMC Research Notes*.

[25] Chekol F. A., Melak M. F., Belew A. K., Zeleke E. G. (2019). Food handling practice and associated factors among food handlers in public food establishments, Northwest Ethiopia. *BMC Research Notes*.

[26] Abdi A. M., Amano A., Abrahim A., Getahun M., Ababor S., Kumie A. (2020). Food hygiene practices and associated factors among food handlers working in food establishments in the Bole Sub City, Addis Ababa, Ethiopia. *Ethiopia. Risk Management and Healthcare Policy*.

[27] Reta M. A., Lemma M. T., Gemeda A. A., Lemlem G. A. (2019). Food handling practice and associated factors among food handlers working in food establishments in Woldia town, Northeast Ethiopia. *Research Square*.

[28] Bafa T. A., Sherif E. M., Hantalo A. H., Woldeamanuel G. G. (2019). Magnitude of enteropathogens and associated factors among apparently healthy food handlers at Wolkite University Student’s cafeteria, southern Ethiopia. *BMC Research Notes*.

[29] Al-Khamees N. A. (2007). Food safety knowledge and reported behaviour of university students in Kuwait. *International Journal of Health Promotion and Education*.

[30] Nahida A. (2007). *Knowledge, Attitude and Practice of Dengue Fever Prevention among the People in Male*.

[31] Angolo C. M. (2011). *Food Safety Knowledge, Beliefs and Self-Reported Handling Practices of International College Students at a Midwestern University*.

[32] Byrd-Bredbenner C., Wheatley V., Schaffner D., Bruhn C., Blalock L., Maurer J. (2007). Development of food safety psychosocial questionnaires for young adults. *Journal of Food Science Education*.

[33] Green E. J., Knechtges P. L. (2015). Food safety knowledge and practices of young adults. *Journal of Environmental Health*.

[34] Ferk C. C., Calder B. L., Camire M. E. (2016). Assessing the food safety knowledge of university of Maine students. *Journal of Food Science Education*.

[35] Moy F. M., Jani R., Halim H. A., Low W. Y. (2018). Determinants of self-reported food safety practices among youths. *British Food Journal*.

[36] Jahed G., GolestaniFar H., Ghodsi R., Mohammadi M. (2012). The knowledge and attitude of students in relation with health and food safety at Tehran university of medical sciences. *Journal of Research and Health*.

[37] Abolhassani M., Eftekhari N., Basirinezhad M. H., Karimi A., Shamsizadeh M. (2018). Knowledge and attitude of students in Shahroud University of Medical Sciences on Health and Food Safety. *Journal of Clinical and Basic Research*.

[38] Yasemin A., Huseyin G., Isil S. (2017). University students’ knowledge and practices of food safety. *The Anthropologist*.

[39] Tavakoli H., Zeynali M., Mehrabi T. A. (2009). Scrutiny of food-borne botulism intoxication in Iran during 2003-2007 with the food hygiene view point. *Hakim Research Journal*.

[40] Mekasha T., Neela S., Kumela D. (2016). Food safety knowledge, practice and attitude of food handlers in traditional hotels of Jimma Town, southern Ethiopia. *Annals Food Science and Technology*.

[41] Nesbitt A., Majowicz S., Finley R. (2009). High-risk food consumption and food safety practices in a Canadian community. *Journal of Food Protection*.

[42] Zyoud S., Shalabi J., Imran K. (2019). Knowledge, attitude and practices among parents regarding food poisoning: a cross-sectional study from Palestine. *BMC Public Health*.

[43] Çakıroğlu F. P., Uçar A. (2008). Employees' perception of hygiene in the catering industry in Ankara (Turkey). *Food Control*.

[44] Lambiri M., Mavridou A., Papadakis J. (2016). The application of hazard analysis critical control point (HACCP) in a flight catering establishment improved the bacteriological quality of meals. *Journal of the Royal Society of Health*.

[45] Salhadi N. A., Ab Hamid M. R., Osman N. S., Nor N. M. (2017). A qualitative study on the determination of healthy cafeteria practice in Selangor, Malaysia. *Malaysia. Environment-Behaviour Proceedings Journal*.

[46] Jeinie M. H., Nor N. M., Sharif M. S. M. (2015). A conceptual model of food hygiene and safety: implication for future research. *Procedia-Social and Behavioral Sciences*.

[47] Vo T. H., Le N. H., Le A. T. N., Minh N. N. T., Nuorti J. P. (2015). Knowledge, attitudes, practices and training needs of food-handlers in large canteens in southern Vietnam. *Food Control*.

[48] Schmidt R. H., Rodrick G. E. (2003). *Food Safety Handbook*.

[49] Al-Shabib N. A., Mosilhey S. H., Husain F. M. (2016). Cross-sectional study on food safety knowledge, attitude and practices of male food handlers employed in restaurants of King Saud University, Saudi Arabia. *Saudi Arabia. Food Control*.

[50] McSwane D. Z. (2004). *Food Safety Fundamentals*.

[51] Bows J., Patrick M., Hale M., Nott K., Hall L. Measurement of three-dimensional temperature distributions and spatial heterogeneity in microwave heated foods by magnetic resonance imaging.

[52] Patah M. O. R. A., Issa Z. M., Nor K. M. (2009). Food safety attitude of culinary arts based students in public and private higher learning institutions (IPT). *International Education Studies*.

[53] Ali A., Angelene P. (2018). Self-reported association and determinants of KAP on food safety and hygiene among private university students in Kedah state, Malaysia. *MOJ Bioequivalence & Bioavailability*.

[54] Abdullahi A., Hassan A., Kadarman N., Saleh A., YuSa B., Lua P. L. (2016). Food safety knowledge, attitude, and practice toward compliance with abattoir laws among the abattoir workers in Malaysia. *International Journal of General Medicine*.

[55] Asmawi U. M. M., Norehan A. A., Salikin K., Rosdi N. A. S., Munir N. A. T. A., Basri N. B. M. (2018). An assessment of knowledge, attitudes and practices in food safety among food handlers engaged in food courts. *Current Research in Nutrition and Food Science Journal*.

[56] Eslami H., Marzban A., AkramiMohajeri F., Rezaei Z., Rafati F. M. (2015). Students' knowledge and attitude of hygiene and food safety at Shahid Sadoughi University of Medical Sciences in Yazd, Iran. *Journal of Community Health Research*.

[57] Akbari N., Azami-Aghdash S., Sohraby-Silabi Y., Jafarzadeh D., Aghdash H. M. (2018). Assessment of students’ attitude and knowledge about food safety and hygiene in Isfahan University of Medical Sciences. *Journal of Food Safety and Hygiene*.

[58] Bourouiba L. (2016). A sneeze. *New England Journal of Medicine*.

[59] Asadi S., Wexler A. S., Cappa C. D., Barreda S., Bouvier N. M., Ristenpart W. D. (2019). Aerosol emission and superemission during human speech increase with voice loudness. *Scientific Reports*.

